# Validation of the Thai Assessment of Criteria for Specific Internet-use Disorders (ACSID-11) among young adults

**DOI:** 10.1186/s12888-023-05210-z

**Published:** 2023-11-08

**Authors:** Yung-Ning Yang, Jian-An Su, Apiradee Pimsen, Jung-Sheng Chen, Marc N. Potenza, Amir H. Pakpour, Ji-Kang Chen, Wai Chuen Poon, Ira Nurmala, Kamolthip Ruckwongpatr, Chung-Ying Lin

**Affiliations:** 1 Department of Pediatrics, E-Da Hospital, I-Shou University, Kaohsiung, Taiwan; 2 School of Medicine, I-Shou University, Kaohsiung, Taiwan; 3 Department of Psychiatry, Chiayi Chang Gung Memorial Hospital, Chiayi, Taiwan; 4 School of Medicine, Chang Gung University, Taoyuan, Taiwan; 5 Department of Nursing, Chang Gung Institute of Technology, Taoyuan, Taiwan; 6 Department of Nursing, College of Medicine, National Cheng Kung University, Tainan, Taiwan; 7 Faculty of Nursing, Mahidol University, Bangkok, Thailand; 8 Department of Medical Research, E-Da Hospital, I-Shou University, Kaohsiung, 824005 Taiwan; 9 Department of Psychiatry, Yale School of Medicine, 300 George St., Suite 901, New Haven, CT 06511 USA; 10 Connecticut Mental Health Center, 34 Park St, New Haven, CT 06519 USA; 11 Connecticut Council On Problem Gambling, 100 Great Meadow Rd., Suite 704, Wethersfield, CT 06109 USA; 12 Child Study Center, Yale School of Medicine, 350 George St, New Haven, CT 06511 USA; 13 Department of Neuroscience, Yale University, New Haven, CT USA; 14 Wu Tsai Institute, Yale University, 200 South Frontage Rd., SHM C-303, New Haven, CT 06510 USA; 15 Department of Nursing, School of Health and Welfare, Jönköping University, Jönköping, Sweden; 16 Department of Social Work, Chinese University of Hong Kong, Shatin, New Territories Hong Kong; 17 Sunway Business School, Sunway University, Selangor, Malaysia; 18 Department of Epidemiology, Faculty of Public Health, Biostatistics, Population Studies and Health Promotion, Universitas Airlangga, Surabaya, Indonesia; 19 Institute of Allied Health Sciences, College of Medicine, National Cheng Kung University, Tainan, Taiwan; 20 University of Religions and Denominations, Qom, Iran; 21 Department of Occupational Therapy, College of Medicine, National Cheng Kung University, Tainan, 701401 Taiwan; 22 Biostatistics Consulting Center, National Cheng Kung University Hospital, College of Medicine, National Cheng Kung University, Tainan, 701401 Taiwan

**Keywords:** Internet addiction, Addictive behaviors, Factor analysis, Young adults, Specific internet use disorder, Psychometric validation

## Abstract

**Background:**

The Assessment of Criteria for Specific Internet-use Disorders (ACSID-11) is a consistent and comprehensive instrument to assess symptoms of specific internet-use disorders including those related to gaming, shopping, pornography use disorder, social networks use and gambling considering criteria in the eleventh revision of the International Classification of Diseases (ICD-11). However, to date, there is little evidence supporting instruments assessing major types of specific internet use disorders in Thailand. The aim of this present study was to assess the psychometric properties of the ACSID-11 among Thai young adults.

**Methods:**

A total of 612 participants were recruited. A confirmatory factor analysis (CFA) examined construct validity of the ACSID-11. Cronbach’s α and McDonald’s ω were used to assess reliability of the ACSID-11. Pearson correlations examined relationships between ACSID-11 domains and Internet Gaming Disorder Scale—Short Form (IGDS9-SF) scores.

**Results:**

The CFA supported validity of the Thai version of the ACSID-11 and a four-factor structure. Specific domains of the Thai ACSID-11, particularly gaming, were positively and significantly correlated with IGDS9-SF scores.

**Conclusions:**

Data indicate that the Thai version of the ACSID-11 is a valid and reliable instrument to assess major types of specific internet use disorders. Additional studies are needed to further examine the validity and reliability of the Thai ACSID-11.

## Introduction

The internet has become an essential part of people’s everyday life and an important vehicle for work, school, and entertainment [1]. Thailand ranked third in internet use in Southeast Asia in 2019 [1], with Thailand showing high levels of internet usage in 77.8% of its total population [2, 3]. Thai people often engage in multiple internet activities such as social networks (29.5%), online shopping (24.6%), and online gaming (18.6%) [2–4]. Additionally, Thailand has ranked 17th globally regarding use of online pornography in 2019 [5]. Thus, internet activities have become important for Thai people for communication, obtaining information, and leisure [2, 3]. To the best of the present authors’ knowledge, few studies have examined these and other online activities (shopping, gambling, pornography use, and social networking use) in Thailand or the extent to which addictive engagement may be involved. A recent Thai study highlighted the need for assessments aimed at understanding specific online activities and disorders [6]. Validating instruments for assessing online activities in Thailand is important for healthcare providers and public health efforts to screen for internet-use disorders.

Over the past several decades, behavioral addictions have been formally recognized [7–11], although multiple proposed conditions (e.g., internet addiction (IA), smartphone addiction, shopping/buying disorder, social networks disorder) are not formally recognized as disorders in main psychiatric nomenclature systems. IA has been proposed as a behavioral addiction with poorly controlled use of the internet leading to adverse consequences being a central feature [12, 13]. Significant concerns regarding IA’s negative consequences and related public health issues have arisen [14, 15]. In 2013, the American Psychiatric Association (APA) has proposed internet gaming disorder (IGD) as a potential disorder in the Diagnostic and Statistical Manual of Mental Disorders, Fifth Edition (DSM-5) [16]. According to the diagnostic criteria outlined in the DSM-5, IGD is characterized by impaired control over gaming of at least 12 months duration that has led to clinically relevant functional impairment [17, 18]. Subsequently, gaming disorder (GD) has also been defined by the World Health Organization (WHO) in the ICD-11 [1, 19]. GD is characterized by persistent gaming behavior and impaired control over gaming, increased priority given to gaming over other activities, and continuation/escalation of gaming despite the occurrence of negative consequences [20]. Additionally, functional impairment (in personal, familial, social or other domains due to gaming) is important [21]. A Delphi study supported the ICD-11 guidelines for GD [22]. The inclusion of GD in the ICD-11 should facilitate prevention, treatment, and public health efforts [23, 24].

Although multiple instruments exist for screening and evaluation of GD, they have limitations related to use of different cut-off scores, assessment of disorders as currently defined by nomenclature systems, and variable testing of psychometric properties [25, 26]. Furthermore, some online activities (i.e., online sexual behaviors, social network use, and shopping) may be considered as compulsive, with some not currently defined in nomenclature systems [26, 27], although there may exist ways for diagnosing such concerns [28]. Such problematic online behaviors might be associated with GD or each other [29–31]. Given such convergences and the public health implications [32–34], a valid psychometric instrument for assessing a range of problematic online activities is important.

It has been proposed that IA should be assessed using two conceptual structures relating to generalized and specific forms [35]. Generalized IA relates to excessive internet use overall while specific IA refers to specified online activities (e.g., involving social networks, gambling, gaming, pornography, or shopping) [36–38]. Griffiths [39] suggested that the internet might act as mediator dependent on context, and both generalized and specific forms of IA warrant assessment.

Müller et al. developed the Assessment of Criteria for Specific Internet-use Disorders (ACSID-11), considering specific internet-based activities related to gaming, gambling, pornography, social networks and shopping [30]. The ACSID-11 showed good validity and high reliability for assessing main types of specific internet-use disorders which are based on ICD-11 diagnostic guidelines for GD, although additional research was recommended [30]. To date, while there exist some Thai instruments assessing online activities like social media use (e.g., the Thai Bergen Facebook Addiction Scale (Thai-BFAS) and Thai-Social Media Engagement Scale (T-SMES) [40, 41]), validating Thai instruments for additional types of potential internet-use disorders is relevant and important for healthcare providers, especially as healthcare systems adopt the ICD-11.

Regarding potential cultural influences, Thai sports (e.g., Muay Thai or a traditional material art, football) are important [42]. Electronic sports (eSports or competitive video gaming) is a new activity supported by the Thai government. Accordingly, most Thai people are inclined to watch sports online (i.e., Live scores), and this may link to online gambling [6, 42–44]. Moreover, a prior review indicates the cultural differences in porn use between Asian people (including Thai people) and other ethnicity populations [45]. Therefore, the ACSID-11 may help assess addictive behaviors involving gaming and gambling in Thai cultures.

Additionally, to date, there are various self-report psychometric instruments assessing IGD, based on the nine IGD DSM-5 criteria (e.g., Internet Gaming Disorder Scale-Short Form or IGDS9-SF) [46]. Some assess GD based on ICD-11 criteria (e.g., Gaming Disorder Test and Gaming Disorder Scale for Young Adults) [47–49]. Because these instruments solely focus on internet gaming or gaming, they can only screen general and overall severity level for IGD or GD symptom experiences [30]. The ACSID-11 is a different psychometric instrument assessing two response types (i.e., frequency and intensity ratings) of GD symptoms [30]. Accordingly, the ACSID-11 may precisely investigate individuals who have risk for developing GD relative to frequency and intensity of gaming [30].

This study aimed to translate the ACSID-11 into Thai and to validate the Thai version of the ACSID-11 via evaluating its psychometric properties, including factor analysis. We hypothesized that the ACSID-11 would show a four-factor structure and be a satisfactory valid and reliable instrument for measuring potential specific internet-use disorders among Thai young adults. Moreover, domains of the ACSID-11 would correlate with other measurements assessing related constructs. Specifically, the ACSID-11 GD domain and IGDS9-SF scores would have significant correlations, while other ACSID-11 domains showing weak or no relationships to IGDS9-SF scores.

## Methods

### Participants and procedure

A convenience sample of 612 university students, with 444 females, was recruited from various universities located in central, northern, and southern regions of Thailand. Participant recruitment and data collection were completed between September 2022 and January 2023. The eligibility criteria were 1) age ≥ 18 years; 2) could understand and read Thai language; 3) enrolled at a university in Thailand (i.e., undergraduates and postgraduates). Participants were recruited via an online survey link and QR code from *SurveyMonkey* through *Facebook* and a university forum by research assistants involved in this study. Because the online survey link was distributed using *Facebook*, the response rate could not be calculated (i.e., there was no information collected regarding how many participants were invited). However, *SurveyMonkey* showed that 152 participants disagreed to participate in the study and 142 participants did not complete the entire survey, possibly as there was no incentive for participation. Participants completed online questionnaires that assessed demographics, internet gaming (IGDS9-SF), and internet-use behaviors/disorders (ACSID-11) and took approximately 10 – 15 min. Before participants responded and agreed to participate, they were informed of the study objectives and provided informed consent. The Human Research Ethics of National Cheng Kung University approved the study (NCKU HREC-E-110–486-2).

This study was granted permission from Professor Matthias Brand for translation of the ACSID-11 into Thai. We translated the ACSID-11 using a standard process [50]. First, two independent Thai-English researchers (i.e., in sport sciences and nursing) translated the questionnaire into Thai. Both forward translations were checked, discussed, and consolidated into one forward translation. Second, two independent bilingual linguists fluent in Thai and English made two backward translations from one forward translation into an English version. Then, three experts (i.e., two nurses and one psychologist) convened and evaluated the consistency of conceptual and linguistic elements between the original version and all translations (i.e., three forward and backward translations) to confirm the Thai version of the ACSID-11.

## Measures

### Demographic information

All participants were asked information regarding their age, gender, self-reported weight and height, any condition or disease during the survey, academic level, and daily hours spent gaming online.

### Assessment of Criteria for Specific Internet-use Disorders (ACSID-11)

The ACSID-11 was used to measure specific internet-use disorders, based on ICD-11 criteria for disorders due to addictive behaviors [30]. This questionnaire assesses multiple activities on the internet (i.e., gaming, shopping, pornography use, social networks use, and gambling) during the previous year [30]. The ACSID-11 instrument includes 11 items categorized into three main criteria (i.e., impaired control (IC), increased priority given to the online activity (IP), continuation/escalation (CE)) with three items each and a fourth domain with two additional items (i.e., functional impairment in daily life and marked distress). Participants were first asked about their past-12-month activities on the internet (i.e., gaming, shopping, pornography use, social networks use, and gambling) via ‘yes’ or ‘no’ responses. Then, participants responded to the 11 items for all internet activities that had previously been answered with ‘yes’. An example IC item is, “*In the past 12 months, have you had trouble keeping track of when you started the activity, for how long, how intensely, or in what situation you did it, or when you stopped?*”. An example IP item is, “*In the past 12 months, have you given the activity an increasingly higher priority than other activities?*”. An example CE item is, “*In the past 12 months, have you continued or increased the activity even though it has threatened or caused you to lose a relationship with someone important to you?*”. Functional impairment in daily life was assessed by, “*Thinking about all areas of your life, has your life been noticeably affected by the activity in the past 12 months?*”. Marked distress was assessed by, “*Thinking about all areas of your life, did the activity cause you suffering in the past 12 months?*”. Participants indicated two-part responses for frequency (0 = “never”, 1 = “rarely”, 2 = “sometimes”, 3 = “often”) and intensity (0 = “not at all intense”, 1 = “rather not intense”, 2 = “rather intense”, 3 = “intense”) per item for each activity. Final scores were calculated by summing the total relevant items in each domain and overall, with higher scores reflecting greater symptom severity for each activity [30]. The ACSID-11 has demonstrated validity and reliability for measuring possible internet use disorders with good internal consistency for both frequency (α = 0.90 – 0.95) and intensity (α = 0.89 – 0.94) ratings in its German version [30]. The original validation study revealed that all items of the ACSID-11 demonstrated an excellent fit with a four-factor structure which was supported by confirmatory factor analysis (CFA) when compared to a unidimensional structure [30]. All four-factor structures of the ACSID-11 reflect ICD criteria for disorders due to addictive behaviors [30]. The ACSID-11 also demonstrated good reliability in the current sample (α = 0.82 – 0.86 for frequency rating; α = 0.87 – 0.95 for intensity rating).

### Internet Gaming Disorder Scale—Short Form (IGDS9-SF)

The IGDS9-SF, based on the nine DSM-5 IGD criteria [48], measured IGD severity. The IGDS9-SF assesses both online and/or offline gaming during the previous year [51]. The 9 items use a five-point Likert type scale (1 = “never”, 2 = “rarely”, 3 = “sometimes”, 4 = “often”, 5 = “very often”). A final score is calculated by summing totals for the nine items, with higher scores reflecting greater IGD severity [52]. An example item is, “*Do you feel preoccupied with your gaming behavior?*”. The IGDS9-SF has been translated into multiple languages and has demonstrated acceptable validity and reliability in, for example, English (α = 0.94) [51], Turkish (α = 0.89) [53], and Chinese (α = 0.94) versions [54]. The Thai IGDS9-SF used in the present study was translated using standard procedures (i.e., forward translation, back translation, and reconciliation) but had not yet been formally examined for validity in Thailand. Therefore, some initial psychometric properties of the Thai IGDS9-SF using the present sample are briefly reported here: Cronbach’s α = 0.87; unidimensionality is supported by the CFA with fit indices of comparative fit index (CFI) = 0.986, Tucker-Lewis index (TLI) = 0.981, root mean square error of approximation (RMSEA) = 0.033, and standardized root mean square residual (SRMR) = 0.078.

### Statistical analysis

All statistical analyses were conducted using Jeffrey's Amazing Statistics Program (JASP) version 0.16.3 [55]. Descriptive analyses were used to examine the characteristics of participants and mean scores of the ACSID-11 and IGDS9-SF. Skewness and kurtosis were examined to determine whether ACSID-11 scores were normally distributed. Most ACSID-11 items (including those assessing gaming, shopping, pornography use, social networks use and online gambling) presented low means and had positive skewness and kurtosis values (Table 2). All ACSID-11 items were examined using factor loadings derived from CFA and the corrected item-total correlation, with a recommended value above 0.4 reflecting acceptability [56, 57]. CFA was used to examine factor structure, using diagonally weighted least square (DWLS) estimation [58, 59]. To examine internal consistency, Cronbach’s α and McDonald’s ω coefficients were used, with the recommended value above 0.7 indicating acceptability [60, 61]. For CFA, we used χ^2^ statistics, the CFI, TLI, SRMR, and RMSEA to examine goodness of fit indices. Model fit was indicated by non-significant χ^2^, CFI > 0.9, TLI > 0.9, RMSEA < 0.08, and SRMR < 0.08 [62, 63].

Lastly, convergent validity was determined by using Pearson correlations to examine relationships between ACSID-11 gaming scores, IGDS9-SF scores, and daily hours spent gaming online with the recommended values of |r|= 0.10–0.30 reflecting small effects, |r|= 0.30–0.50 reflecting medium effects, and |r|> 0.50 reflecting large effects [64]. According to previous studies, the IGDS9-SF is a valid instrument for assessing IGD [46, 51]. We therefore investigated convergent validity between gaming concerns assessed using the ACSID-11 and IGDS9-SF. Moreover, because the ACSID-11 assesses other online activities that are different from gaming, these distinct activities would likely not correlate strongly with IGDS9-SF scores. Therefore, we also used the IGDS9-SF to assess divergent validity of the ACSID-11 in non-gaming domains.

## Results

According to Table 1, the mean age of participants was 20.57 (SD = 2.29) years with a range between 17 and 33 years. The mean BMI of participants was 21.79 (4.26) kg/m^2^ with a range between 13.84 and 42.06 kg/m^2^. Most participants were female (73%), had no condition/diseases (87%), and were undergraduates (96%). The mean reported daily hours spent gaming online was 1.59 (SD = 1.83) hours with a range between 0 and 12 h. Considerable percentages of participants engaged in gaming (57%), online shopping (80%), online pornography use (41%), social networks use (92%) and online gambling (19%). The mean IGDS9-SF score was 13.16 (SD = 4.83) with a range between 9 and 45. Moreover, participants’ information (i.e., age, BMI, daily hours spent gaming, and IGDS9-SF scores) showed positive skewness and kurtosis. The mean ACSID-11 (including frequency and intensity rating) scores is indicated in Table 2.

**Table 1:** The characteristics of participants (*n* = 612)

	**Min**	**Max**	**Mean (SD)**	**Skewness**	**Kurtosis**	**N (%)**
**Age (years)**	17	33	20.57(2.29)	2.471	8.782	–
**Gender**						
**Male**	-	-	-	-	-	168 (27%)
**Female**	-	-	-	-	-	444 (73%)
**BMI (kg/m**^**2**^**)**	13.84	42.06	21.79(4.26)	1.367	2.473	–
**Any condition or disease**						
Yes	-	-	-	-	-	80(13%)
No	-	-	-	-	-	532(87%)
**Student status**						
Undergraduate	-	-	-	-	-	590(96%)
Postgraduate	-	-	-	-	-	22(4%)
**Daily hours on gaming**	0	12	1.59(1.83)	1.581	3.453	–
**IGDS-SF**	9	45	13.16(4.83)	1.910	5.318	–

*SD *Standard deviation

*BMI *Body Mass Index

*IGDS9-SF *Internet Gaming Disorder Scale—Short Form

**Table 2:** Psychometric properties of the ACSID-11 at the item level

**Gaming** (*n* = 352)														
	**Frequency rating**							**Intensity rating**						
	^**a**^**Factor loadings**	**Item-total correlation**	**Mean (SD)**	**Skewness**	**Kurtosis**	**α**	**ω**	^**a**^**Factor loadings**	**Item-total correlation**	**Mean (SD)**	**Skewness**	**Kurtosis**	**α**	**ω**
Total						0.82	0.82						0.88	0.89
AC-IC						0.65	0.65						0.74	0.75
Item 1	0.59	0.45	1.10(1.02)	0.44	-1.00			0.66	0.57	0.35(0.61)	1.82	3.31		
Item 2	0.56	0.48	0.78(0.92)	0.84	-0.46			0.66	0.58	0.29(0.60)	2.24	4.94		
Item 3	0.73	0.47	0.41(0.76)	1.84	2.59			0.81	0.58	0.18(0.48)	3.09	10.53		
AC-IP						0.79	0.80						0.79	0.80
Item 4	0.75	0.62	0.55(0.82)	1.40	1.13			0.75	0.62	0.24(0.54)	2.62	7.42		
Item 5	0.78	0.69	0.42(0.76)	1.83	2.52			0.79	0.71	0.18(0.48)	2.91	8.57		
Item 6	0.71	0.59	0.34(0.70)	2.19	4.23			0.71	0.59	0.15(0.44)	3.48	13.34		
AC-CE						0.82	0.82						0.82	0.82
Item 7	0.78	0.68	0.25(0.60)	2.73	7.43			0.79	0.68	0.13(0.43)	3.73	15.40		
Item 8	0.80	0.68	0.24(0.58)	2.51	6.00			0.85	0.68	0.11(0.39)	3.95	16.42		
Item 9	0.74	0.65	0.27(0.61)	2.48	6.13			0.78	0.65	0.13(0.42)	3.51	13.71		
AC-FI						0.79	0.79						0.80	0.80
Item 10	0.81	0.66	0.32(0.64)	2.19	4.64			0.84	0.68	0.18(0.49)	2.95	9.05		
Item 11	0.81	0.66	0.25(0.58)	2.57	6.62			0.80	0.68	0.14(0.43)	3.51	14.05		
**Online shopping** (*n* = 493)														
	**Frequency rating**							**Intensity rating**						
	^**a**^**Factor loadings**	**Item-total correlation**	**Mean (SD)**	**Skewness**	**Kurtosis**	**α**	**ω**	^**a**^**Factor loadings**	**Item-total correlation**	**Mean (SD)**	**Skewness**	**Kurtosis**	**α**	**ω**
Total						0.84	0.85						0.87	0.87
AC-IC						0.73	0.74						0.83	0.84
Item 1	0.53	0.51	1.42(0.94)	0.18	-0.84			0.71	0.66	0.42(0.64)	1.50	1.96		
Item 2	0.68	0.60	1.01(0.96)	0.57	-0.69			0.81	0.75	0.36(0.65)	1.97	3.81		
Item 3	0.87	0.55	0.62(0.86)	1.21	0.53			0.86	0.67	0.24(0.55)	2.62	7.30		
AC-IP													0.82	0.82
Item 4	0.78	0.62	0.64(0.84)	1.16	0.47	0.81	0.81	0.82	0.62	0.25(0.55)	2.58	7.54		
Item 5	0.77	0.70	0.47(0.76)	1.51	1.43			0.79	0.74	0.18(0.46)	2.99	10.04		
Item 6	0.75	0.67	0.45(0.76)	1.63	1.79			0.71	0.69	0.15(0.43)	3.25	12.60		
AC-CE													0.86	0.86
Item 7	0.86	0.79	0.34(0.68)	2.07	3.71	0.89	0.89	0.79	0.72	0.12(0.41)	4.03	19.19		
Item 8	0.87	0.81	0.31(0.64)	2.22	4.53			0.85	0.77	0.13(0.42)	3.60	14.20		
Item 9	0.84	0.76	0.33(0.67)	2.08	3.78			0.82	0.71	0.13(0.42)	3.65	15.54		
AC-FI													0.84	0.84
Item 10	0.88	0.75	0.42(0.73)	1.74	2.37	0.86	0.86	0.88	0.73	0.21(0.51)	2.80	8.64		
Item 11	0.85	0.75	0.35(0.69)	2.00	3.37			0.83	0.73	0.16(0.46)	3.40	13.07		
**Online pornography use** (*n* = 250)														
	**Frequency rating**							**Intensity rating**						
	^**a**^**Factor loadings**	**Item-total correlation**	**Mean (SD)**	**Skewness**	**Kurtosis**	**α**	**ω**	^**a**^**Factor loadings**	**Item-total correlation**	**Mean (SD)**	**Skewness**	**Kurtosis**	**α**	**ω**
Total						0.82	0.82						0.88	0.88
AC-IC						0.76	0.77						0.84	0.85
Item 1	0.69	0.57	0.54(0.81)	1.46	1.38			0.72	0.68	0.23(0.55)	2.77	8.50		
Item 2	0.68	0.64	0.45(0.84)	1.90	2.64			0.79	0.77	0.19(0.55)	3.32	11.76		
Item 3	0.80	0.60	0.28(0.65)	2.61	6.59			0.91	0.69	0.14(0.47)	4.02	17.45		
AC-IP						0.78	0.79						0.88	0.89
Item 4	0.77	0.60	0.25(0.62)	2.69	7.13			0.86	0.77	0.14(0.49)	4.09	17.95		
Item 5	0.77	0.68	0.25(0.64)	2.74	7.17			0.86	0.82	0.13(0.47)	4.18	18.78		
Item 6	0.68	0.59	0.22(0.60)	3.09	9.45			0.82	0.73	0.10(0.40)	4.97	27.44		
AC-CE						0.88	0.88						0.89	0.90
Item 7	0.86	0.79	0.16(0.50)	3.61	13.41			0.88	0.76	0.08(0.36)	5.14	29.49		
Item 8	0.78	0.75	0.14(0.46)	3.88	16.29			0.84	0.80	0.08(0.36)	5.64	36.00		
Item 9	0.89	0.77	0.15(0.47)	3.66	14.33			0.84	0.81	0.09(0.38)	5.13	29.45		
AC-FI						0.87	0.87						0.84	0.84
Item 10	0.86	0.76	0.16(0.505)	3.64	14.31			0.88	0.72	0.10(0.40)	4.61	23.67		
Item 11	0.88	0.76	0.14(0.47)	3.74	14.81			0.82	0.72	0.08(0.35)	5.14	30.43		
**Social networks use** (*n* = 568)														
	**Frequency rating**							**Intensity rating**						
	^**a**^**Factor loadings**	**Item-total correlation**	**Mean (SD)**	**Skewness**	**Kurtosis**	**α**	**ω**	^**a**^**Factor loadings**	**Item-total correlation**	**Mean (SD)**	**Skewness**	**Kurtosis**	**α**	**ω**
Total						0.86	0.87						0.88	0.88
AC-IC						0.70	0.71						0.81	0.82
Item 1	0.42	0.39	2.19(1.05)	-0.99	-0.40			0.73	0.67	0.72(0.89)	1.07	0.21		
Item 2	0.67	0.60	1.34(1.07)	0.16	-1.23			0.73	0.73	0.51(0.74)	1.47	1.74		
Item 3	0.87	0.54	0.91(1.05)	0.76	-0.75			0.83	0.63	0.35(0.65)	2.02	4.00		
AC-IP						0.83	0.84						0.85	0.85
Item 4	0.75	0.65	1.10(1.08)	0.47	-1.12			0.82	0.69	0.46(0.74)	1.65	2.23		
Item 5	0.82	0.75	0.78(1.02)	0.98	-0.39			0.82	0.77	0.31(0.61)	2.11	4.33		
Item 6	0.81	0.69	0.69(0.97)	1.14	0.01			0.79	0.71	0.28(0.59)	2.40	6.04		
AC-CE						0.87	0.87						0.85	0.86
Item 7	0.85	0.75	0.52(0.91)	1.68	1.61			0.81	0.73	0.20(0.53)	3.01	9.98		
Item 8	0.85	0.78	0.55(0.92)	1.55	1.21			0.85	0.77	0.23(0.55)	2.69	7.52		
Item 9	0.79	0.72	0.60(0.94)	1.40	0.71			0.77	0.68	0.25(0.54)	2.44	6.37		
AC-FI						0.87	0.87						0.81	0.82
Item 10	0.87	0.77	0.67(0.95)	1.19	0.24			0.91	0.69	0.34(0.63)	2.01	4.06		
Item 11	0.88	0.77	0.57(0.93)	1.47	0.93			0.76	0.69	0.25(0.57)	2.61	7.33		
**Online gambling** (*n* = 113)														
	**Frequency rating**							**Intensity rating**						
	^**a**^**Factor loadings**	**Item-total correlation**	**Mean (SD)**	**Skewness**	**Kurtosis**	**α**	**ω**	^**a**^**Factor loadings**	**Item-total correlation**	**Mean (SD)**	**Skewness**	**Kurtosis**	**α**	**ω**
Total						0.83	0.82						0.95	0.95
AC-IC						0.54	0.55						0.79	0.79
Item 1	0.64	0.37	0.09(0.37)	4.89	27.26			0.75	0.69	0.09(0.40)	5.41	31.44		
Item 2	0.40	0.37	0.22(0.70)	3.28	9.55			0.66	0.59	0.11(0.49)	4.89	24.12		
Item 3	0.69	0.41	0.10(0.42)	4.93	26.03			0.86	0.64	0.09(0.42)	5.49	31.51		
AC-IP						0.67	0.70						0.90	0.90
Item 4	0.69	0.40	0.08(0.36)	5.62	35.25			0.88	0.75	0.08(0.41)	5.85	35.22		
Item 5	0.55	0.57	0.10(0.43)	5.04	26.75			0.90	0.87	0.07(0.37)	6.38	42.41		
Item 6	0.60	0.52	0.12(0.54)	4.60	20.53			0.80	0.78	0.07(0.40)	5.99	36.89		
AC-CE						0.87	0.87						0.92	0.93
Item 7	0.78	0.74	0.06(0.33)	6.48	44.67			0.89	0.85	0.06(0.36)	6.37	42.46		
Item 8	0.85	0.73	0.05(0.29)	6.80	51.97			0.93	0.88	0.07(0.38)	6.04	37.73		
Item 9	0.85	0.78	0.06(0.32)	6.16	41.17			0.87	0.81	0.06(0.35)	6.46	44.34		
AC-FI						0.68	0.68						0.87	0.87
Item 10	0.79	0.53	0.08(0.38)	5.95	37.98			0.87	0.77	0.06(0.35)	6.55	45.85		
Item 11	0.67	0.53	0.05(0.31)	7.08	54.78			0.89	0.77	0.06(0.35)	6.65	47.04		

^a^Factor loadings derived from Confirmatory Factor Analysis

*ACSID-11* Assessment of Criteria for Specific Internet-use Disorders

*AC-IC* Assessment of Criteria for Specific Internet-use Disorders (impaired control domain score)

*AC-IP* Assessment of Criteria for Specific Internet-use Disorders (increased priority given to the online activity domain score)

*AC-CE* Assessment of Criteria for Specific Internet-use Disorders (continuation/escalation domain score)

*AC-FI* Assessment of Criteria for Specific Internet-use Disorders (functional impairment domain in daily life and marked distress score)

*SD* Standard deviation

*a* Cronbach alpha coefficient

Ω McDonald omega coefficient

CFA supported the four-factor structure of the ACSID-11 (see fit indices in Tables 2 and 3). The ACSID-11 showed good fit of both frequency and intensity ratings with non-significant χ^2^and values of model fit (i.e., CFI, RMSEA, and SRMR) achieving the suggested cut-offs. Moreover, all types of specific internet use disorders assessed by the ACSID-11 (including frequency and intensity ratings) demonstrated acceptable standardized factor loadings and item-total correlations. Moreover, the internal consistency was acceptable and satisfactory for the four-factor structure including frequency and intensity ratings except for the IC domain of gaming in the frequency rating (both Cronbach’s α and McDonald’s ω = 0.65), the IC domain of online gambling in the frequency rating (Cronbach’s α = 0.54 and McDonald’s ω = 0.55), the IP domain of online gambling in the frequency rating (Cronbach’s α = 0.67) and the FI domain of online gambling in the frequency rating (both Cronbach’s α and McDonald’s ω = 0.68).

**Table 3:** Index of fit in the confirmatory factor analysis of the ACSID-11

		**Frequency rating**						**Intensity rating**					
		**χ**^**2**^** (*df*)**	***p*-value**	**CFI**	**TLI**	**RMSEA (90%CI)**	**SRMR**	**χ**^**2**^** (*df*)**	***p*-value**	**CFI**	**TLI**	**RMSEA (90%CI)**	**SRMR**
**Gaming**	**a**	86.61(44)	< .001	0.983	0.979	0.040 (0.027,0.052)	0.063	26.75(44)	0.981	1.000	1.014	0.000 (0.000,0.000)	0.057
	**b**	29.12(38)	0.849	1.000	1.005	0.000 (0.000,0.016)	0.034	10.03(38)	1.000	1.000	1.027	0.000 (0.000,0.000)	0.034
**Online shopping**	**a**	128.18(44)	< .001	0.976	0.970	0.056 (0.045,0.067)	0.076	39.31(44)	0.673	1.000	1.004	0.000 (0.000,0.022)	0.076
	**b**	41.18(38)	0.333	0.999	0.999	0.012 (0.000,0.031)	0.042	11.73(38)	1.000	1.000	1.025	0.000 (0.000,0.000)	0.043
**Online pornography use**	**a**	62.81(44)	0.033	0.988	0.985	0.026 (0.008,0.040)	0.089	22.36(44)	0.997	1.000	1.032	0.000 (0.000,0.000)	0.084
	**b**	14.53(38)	1.000	1.000	1.022	0.000 (0.000,0.000)	0.039	5.56(38)	1.000	1.000	1.056	0.000 (0.000,0.000)	0.042
**Social networks use**	**a**	146.53(44)	< .001	0.980	0.975	0.062 (0.051,0.073)	0.070	60.20(44)	0.053	0.994	0.993	0.025 (0.000,0.039)	0.072
	**b**	51.38(38)	0.072	0.997	0.996	0.024 (0.000,0.039)	0.039	18.66(38)	0.996	1.000	1.010	0.000 (0.000,0.000)	0.038
**Online gambling**	**a**	18.98(44)	1.000	1.000	1.093	0.000 (0.000,0.000)	0.068	3.70(44)	1.000	1.000	1.097	0.000 (0.000,0.000)	0.048
	**b**	8.68(38)	1.000	1.000	1.126	0.000 (0.000,0.000)	0.047	1.929(38)	1.000	1.000	1.100	0.000 (0.000, 0.000)	0.034

^a^Assessment of Criteria for Specific Internet-use Disorders (one-factor structure)

^b^Assessment of Criteria for Specific Internet-use Disorders (four-factor structure)

*CFI* Comparative fit index

*TLI* Tucker-Lewis index

*RMSEA* Root mean square error of approximation

*SRMR* Standardized root mean square residual

Most of the four ACSID-11 domains (i.e., IC, IP, CE, and FI) were positively correlated with IGDS9-SF scores, showing small to moderate effects (Table 4). For gaming, all four ACSID-11 domains (including frequency and intensity ratings) correlated with IGDS9-SF scores and gaming time, showing moderate effects. For online shopping, only the FI domain of frequency ratings and all domains of intensity ratings correlated with IGDS9-SF scores, showing small effects. For online pornography, most ACSID-11 domains (including frequency and intensity ratings) correlated with IGDS9-SF scores and gaming time, showing small effects. For social networks use, all four ACSID-11 domains (including frequency and intensity ratings) correlated with IGDS9-SF scores, showing small effects, except for the IC domain of frequency ratings; and only the CE domain of intensity ratings of the ACSID-11 for social networks use correlated with gaming time. For online gambling, most ACSID-11 domains (including frequency and intensity ratings) correlated with IGDS9-SF scores and gaming time, showing small effects. All ACSID-11 domains for gaming correlated robustly with other ACSID-11-assessed internet use disorders. Moreover, IGDS9-SF scores correlated with gaming time. showing a moderate effect.

**Table 4:** Correlation among ACSID-11 scores, IGDS9-SF scores, and gaming time

		**Frequency rating**				**Intensity rating**			
		**IC**	**IP**	**CE**	**FI**	**IC**	**IP**	**CE**	**FI**
**Gaming**	**IGDS-T**	0.39***	0.40***	0.38***	0.39***	0.43***	0.47***	0.46***	0.45***
	**Gaming time**	0.53***	0.36***	0.33***	0.35***	0.36***	0.30***	0.30***	0.28***
**Online shopping**	**IGDS-T**	-0.06 (0.122)	0.02 (0.602)	0.05 (0.214)	0.12**	0.10*	0.20***	0.21***	0.19***
	**Gaming time**	-0.03 (0.500)	0.01 (0.798)	0.01 (0.751)	0.02 (0.708)	-0.01 (0.892)	0.06 (0.144)	0.06 (0.135)	0.04 (0.335)
**Online pornography use**	**IGDS-T**	0.19***	0.18***	0.23***	0.21***	0.18***	0.22***	0.27***	0.24***
	**Gaming time**	0.24***	0.13**	0.17***	0.15***	0.13**	0.12**	0.17***	0.11**
**Social networks use**	**IGDS-T**	0.02 (0.624)	0.09*	0.08*	0.14***	0.16***	0.20***	0.25***	0.24***
	**Gaming time**	-0.002 (0.968)	-0.002 (0.954)	0.01 (0.856)	-0.01 (0.845)	0.02 (0.658)	0.04 (0.354)	0.08*	0.04 (0.355)
**Online gambling**	**IGDS-T**	0.17***	0.12**	0.21***	0.14***	0.15***	0.18***	0.22***	0.22***
	**Gaming time**	0.14***	0.06	0.08*	0.06	0.07	0.06	0.08*	0.08*
**IGDS-T**	**Gaming time**	0.46***							

^*^ *p* < .05, ** *p* < .01, *** *p* < .001

*ACSID-11* Assessment of Criteria for Specific Internet-use Disorders

*AC-IC* Assessment of Criteria for Specific Internet-use Disorders (Total score of impaired control domain score)

*AC-IP* Assessment of Criteria for Specific Internet-use Disorders (Total score of increased priority given to the online activity domain score)

*AC-CE* Assessment of Criteria for Specific Internet-use Disorders (Total score of continuation/escalation domain score)

*AC-FI* Assessment of Criteria for Specific Internet-use Disorders (Total score of functional impairment in daily life and marked distress domain score)

*IGDS-T* Internet Gaming Disorder Scale—Short Form (Total score)

## Discussion

The present study examined the psychometric properties of the Thai ACSID-11 among Thai young adults. The ACSID-11 appears suitable for assessing multiple types of specific internet-use disorders related to gaming, shopping, pornography use, social networks use and gambling among Thai young adults. Findings supported our hypotheses. Specifically, the ACSID-11, regardless of online activity, had four-factor structures with adequate CFA fit indices. Additionally, standardized factor loadings and item-total correlations were acceptable for both frequency and intensity ratings of the Thai ACSID-11, consistent with the original version of ACSID-11 [30].

Regarding reliability, the Thai ACSID-11 demonstrated acceptable internal consistency including frequency and intensity ratings comparable to the original version [30]. However, the results of the IC domain of gaming (frequency rating) and IC, IP and FI and domains of online gambling (frequency ratings) demonstrated slightly lower internal consistency (Cronbach’s α = 0.54 – 0.68 and McDonald’s ω = 0.55—0.68). We suspect that this might reflect the relatively small number of participants reporting online gaming (*n* = 352) and online gambling (*n* = 113). In this regard, we suggest that future studies should consider a larger sample of people who engage in online gaming and gambling to further validate these specific internet activities assessed with the ACSID-11.

All four gaming domains of the ACSID-11 demonstrated significant correlations with IGDS9-SF scores and time spent gaming. The original ACSID-11 validation study assessed correlations between ACSID-11 and IGDT-10 (Ten-Item Internet Gaming Disorder Test) scores [30], with ACSID-11 and IGDT-10 scores showing positive correlations [30]. Despite using the IGDS9-SF in place of the IGDT-10, results were comparable given that the IGDS9-SF and IGDT-10 assess similar concepts, the IGDS9-SF is a standardized instrument based on DSM-5 criteria for IGD [51], and scores on the IGDT-10 and IGDS9-SF correlate [65]. Importantly, our findings revealed that the gaming domain of the ACSID-11 was moderately correlated with IGDS9-SF scores while other online activities (i.e., shopping, pornography use, social networks use and online gambling) were uncorrelated or modestly correlated with IGDS9-SF scores, at most showing small effects. Taken together, the current findings suggest specificity but also some inter-relationships between IGD and multiple other types of internet-use disorders related to online shopping, online pornography use, social networks use and gambling.

The present results suggest that some domains are not strongly related to IGD. For example, ACSID-11-assessed online shopping (IC, IP, and CE domains) and social networks use (IC domain) in frequency ratings were not correlated with IGDS9-SF scores. These findings suggest distinct relationships and specific entities related to specific types of internet-use disorders. Future studies should focus on factors related to specific types and patterns of internet-use disorders [32].

Study limitations warrant mention. First, participants were Thai university students and were recruited by convenience sampling. Therefore, our sample might not be representative. Second, data collection involved self-reported questionnaires, and are thus susceptible to related biases (e.g., memory, social desirability). Third, the study sample was moderate in size. Larger studies involving different samples (including clinical populations) should be examined to validate further the Thai ACSID-11, especially with respect to gaming and gambling. Fourth, the study was cross-sectional, and future longitudinal studies should assess other features (e.g., test/retest reliability). Finally, the IGDS9-SF was used to measure convergent (for gaming assessed in the ACSID-11) and divergent (for activities other than gaming assessed in the ACSID-11) validity of the ACSID-11. A limitation was that the ACSID-11 was not examined for convergent validity for non-gaming activities. Future research should use other instruments (e.g., Problematic Pornography Consumption Scale [66] or Brief Pornography Screen [67] for ACSID-11 pornography use or the Bergen Social Media Addiction Scale [68] for ACSID-11 social media use) to examine the convergent validity of specific online activities assessed in the ACSID-11.

In conclusion, the present study expands research on validation tools to assess major types of specific internet-use disorders among Thai university students. The Thai ACSID-11 may be used to assess main types of specific internet-use disorders related to gaming, shopping, pornography use, social networks use and gambling. Larger, diverse populations should be considered in future research to examine further the validity and reliability of the Thai ACSID-11.

## Data Availability

The datasets generated during and/or analyzed during the current study are available from the corresponding author on reasonable request.

## Abbreviations

IA: Internet addiction
GD: Gaming disorder
ACSID-11: Assessment of Criteria for Specific Internet-use Disorders
IGDS9-SF: Internet Gaming Disorder Scale—Short Form
IGDT-10: Ten-Item Internet Gaming Disorder Test
CFA: Confirmatory factor analysis
